# Study on Rheological Behavior of Micro/Nano-Silicon Carbide Particles in Ethanol by Selecting Efficient Dispersants

**DOI:** 10.3390/ma13071496

**Published:** 2020-03-25

**Authors:** Guoqiang Luo, Zhuang Zhang, Jianian Hu, Jian Zhang, Yi Sun, Qiang Shen, Lianmeng Zhang

**Affiliations:** State Key Laboratory of Advanced Technology for Materials Synthesis and Processing, Wuhan University of Technology, Wuhan 430070, China; luogq@whut.edu.cn (G.L.); zz1994@whut.edu.cn (Z.Z.); hujianian1@outlook.com (J.H.); zhangjian178@whut.edu.cn (J.Z.); sqqf@263.net (Q.S.); lmzhang@whut.edu.cn (L.Z.)

**Keywords:** rheology, SiC suspension, colloidal stability, dispersion mechanism, flow index factor

## Abstract

A colloidal stability study of a nonaqueous silicon carbide suspension is of great significance for preparing special silicon carbide ceramics by colloidal processing. In this paper, three different chemical dispersants, which are amphiphilic, acidophilic, and alkaliphilic, are selected to compare their ability to stabilize nonaqueous slurries of silicon carbide. The analysis of the flow index factor is first used to estimate the colloidal stability of the suspensions. The results show that the addition of only 5 wt.% polyvinylpyrrolidone (PVP) forms a silicon carbide slurry with a low viscosity value of 17 mPa⋅s at 25 s^−1^. In addition, Fourier transform infrared spectroscopy (FTIR) and X-ray photoelectron spectroscopy (XPS)measurements indicate that the PVP molecule is successfully adsorbed on the surface of silicon carbide. The different adsorption models are fitted, and the adsorption of PVP molecules on the surface of silicon carbide belongs to the Langmuir single-layer adsorption model. At the optimal PVP amount, the volume content of the suspension is as high as 22.27 vol.%, a Newtonian-like fluid still appears, and no agglomerate structure is formed in the system. After the volume content exceeds 22.27 vol.%, the flow index factor of the slurry begins to plummet, indicating that the slurry begins to transform from a Newtonian-like fluid to a shear-thinning fluid. The particles undergo inevitable agglomeration accompanied by the emergence of yield stress. Finally, a maximum solid loading of the system is predicted to be 46 vol.%, using the Krieger-Dougherty model.

## 1. Introduction

Silicon carbide ceramics are emerging candidates for special ceramics owing to their high hardness, high mechanical strength, high antioxidant capacity, and corrosion resistance. Silicon carbide ceramics are widely used in the armor, aerospace, medical, chemical, steel metallurgy, and electronics industries, as well as other fields. However, owing to the high strength and high brittleness of silicon carbide (SiC), the preparation of silicon carbide devices with complex sizes is limited, which increases the economic cost in their subsequent processing [1,2,3,4,5].

As a result, increasing attention has been focused on colloidal processing methods, which can lead to complex shapes and uniform microstructures of green bodies. In recent years, colloidal processing, such as slip casting and tape casting, has been widely used to produce special ceramics from SiC powders [6,7,8,9]. Therefore, it is particularly important to obtain ceramic suspensions with good dispersion stability and reliable rheological behaviors. The colloidal techniques first need to achieve sufficient dispersion of the ceramic particles in the solvent. In particular, a starting suspension with high ceramic loading and low viscosity is also required [10,11,12].

The scale effect of nanopowders offers various advantages, and these powders have attracted widespread attention for their selection as raw powders for molding. However, micro- and nanopowders, owing to their high surface energy, are found to form agglomerated structures in solvents extremely easily, which affects the subsequent processing [13,14]. When using water as a solvent, owing to a large number of hydrogen bonds in the water, additional hydrogen bonds are easily formed between the particles and cause agglomeration. Additionally, the surface tension of water is so high that it is difficult to wet the fine powder, which limits its application in a colloidal process. In contrast, organic solvents have low surface tension, so environmentally-friendly organic solvents have attracted more attention [15,16,17,18].

At present, a large number of studies have focused on achieving good dispersion of silicon carbide powder into water, and the commonly used dispersants are mainly small molecules and polyelectrolytes, such as tetramethylammonium hydroxide (TMAH) and polyethyleneimine (PEI) [19,20,21,22,23,24,25,26,27,28,29,30]. For our own research, tape casting is used to obtain dense SiC ceramics. In terms of solvent options, we chose ethanol as the solvent because, to date, it is difficult to find a suitable binder that can be efficiently dissolved into water; additionally, the colloidal process usually requires the addition of a variety of polymers, which are prone to produce complex effects in water, and we lack certain knowledge about what those interactions are [19,20,21,22,23,24,25,28,29,30,31,32]. Unfortunately, most of these studies were performed in deionized water. Only a few studies have focused on stabilization mechanisms in organic solvents [26,27]. Prasenjit et al. [33] reported a few parameters on the rheological behavior of SiC nanoparticles in an aqueous solvent. However, for the rheological analysis, only the viscosity data of the suspension are analyzed, and no further analysis is made on the change of the fluid form. Moreover, there is no study on the maximum solids content of the suspension, which is an important parameter in practical applications.

Polyvinylpyrrolidone (PVP) has been widely used in current markets. PVP was originally applied as an extension agent for plasma substitutes and was later widely used in diverse fields. PVP is an amphiphilic polymer that is harmless to humans and the environment [34,35,36]. For common organic solvents, such as amines, alcohols, and acids, PVP easily dissolves into them. The polymer also has a long history in the food industry and ceramic processing as a highly efficient multifunctional dispersant.

PEI is an effective dispersant in a low pH suspension. The stability mechanism of PEI in suspension is a combination of steric hindrance and surface charge repulsion mechanisms that has been successfully applied to stabilize different ceramic suspensions [19,20,21,22,23,24]. The ability of PEI to acquire hydrogen ions was affected by the pH value; the protonation (~0.1%) of PEI almost occurred at pH 10, and it was completely protonated at pH 2. PEI is a polyelectrolyte with many branches. During the polymerization process, the polymerization of aziride leads to the formation of PEI, some oligomers are produced, and many tertiary amine groups are present, causing PEI to have a high charge density. This also makes it acidophilic. The high branching properties of PEI allow for a high charge concentration and various molecular weights; therefore, it can also act as a chain-entangled coagulation agent to agglomerate the particles [37].

Tetramethylammonium hydroxide (TMAH, 25% aqueous solution, 91.153 g/mol, Sigma-Aldrich Co, Shanghai, China) has a chemical formula of N(CH3)_4_^+^OH^−^ and is a quaternary ammonium salt. Its stabilizing mechanism is mainly its use as a free electrolyte, which increases the ionic strength and disturbs the electrostatic repulsion between particles; this causes a bridging effect between particles and leads to slight agglomerations [25].

In the present work, the effects of different dispersants, dispersant concentrations, and solids loading on the rheological properties of silicon carbide nonaqueous slurries were investigated. When PVP was used as a dispersing agent, an organic suspension of well-dispersed silicon carbide was prepared. The purpose of this work was to use rheological knowledge to quantitatively evaluate the efficiency of dispersant. The maximum solid loading was estimated by the Einstein and Krieger-Dougherty models. The adsorption mechanism was determined using classical monolayer Langmuir isotherm and multilayer Freundlich isotherm models, and the possible mechanism of dispersant stabilization was also investigated.

## 2. Materials and Methods 

### 2.1. Starting Materials

**Figure 1:**
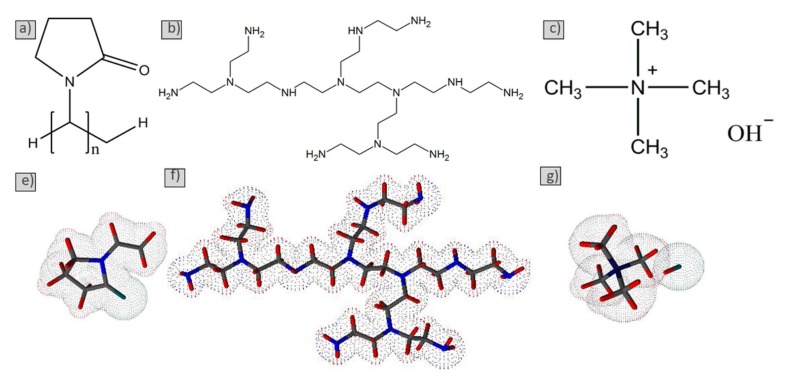
Chemical and partial spatial formula of polyvinylpyrrolidone (PVP) (**a**,**e**), polyethyleneimine (PEI) (**b**,**f**), and tetramethylammonium hydroxide (TMAH) (**c**,**g**).

The powder used was silicon carbide (Aladdin, Shanghai, China, >99% purity), and the average particle size was 303 nm (d50). The average size (d50) of the powder was determined by laser diffraction (Mastersizer 2000, Wuhan University of Technology, Wuhan, China). The crystal structure of raw material powder was identified by X-ray diffraction (XRD) using Cu Kα radiation. (RU-200B/D/MAX-RB, Rigaku, Tokyo Japan). Scanning electron microscopy (SEM, Zeiss Ultra Plus/Zeiss Ultra Plus, Carl Zeiss AG, Shanghai, China) was used to investigate the morphology and size of silicon carbide powders. The technical characteristics of silicon carbide surface chemistry were determined by X-ray photoelectron spectroscopy (XPS) (ESCALAB 250Xi, Thermo Fisher Scientific, Shanghai, China) and Fourier transform infrared spectroscopy (FTIR) (Nicolet 6700, Thermo Electron Scientific Instruments, Shanghai, China). The dispersants used include PVP (Mv 50,000, >99% Shanghai Aladdin Biochemical Technology Co., Ltd, Shanghai, China), polymeric polyethyleneimine (PEI) with molecular weights of 1800 g/mol (Aladdin Industrial Corporation, Shanghai, China), and TMAH (Mv 91.15, 25% aqueous solution, Aladdin Industrial Corporation, Shanghai, China). The structural details of the three dispersants are shown in Figure 1, and the spatial structures were optimized in an Advanced Chemistry Development (ACD) /3D Viewer. An organic solvent of ethanol (≥99.7%) was used as a dispersion medium.

### 2.2. Preparation of SiC Suspensions

The silicon carbide powder was mixed with ethanol, and then dispersant was added. Each dispersant content was from 1 wt.% to 6 wt.% (based on a certain amount of the powder weight), and solids loading of the ceramic suspensions was fixed at 50 wt.% (20 vol.%). The volume fractions of the suspension were calculated using Equation (1):

(1) $$\varphi_{v}=\frac{m_{n}/\rho_{n}}{\left(m_{n}/\rho_{n}\right)+\left(m_{b}/\rho_{b}\right)}$$

where *φ_v_* denotes the volume fraction of suspension (%); *m_n_* and *m_b_* are the weight of nanoparticles and the base medium, respectively; and *ρ_n_* and *ρ_b_* determine the densities of the nanoparticle and base liquid, respectively.

In a practical process, ball milling containing 5 mm yttrium oxide-stabilized zirconia balls (QM-QX0.4, Nanjing Nanda Instrument Co. Ltd, Nanjing, China) was used to mix and deagglomerate suspensions at 300 rpm for 24 h at room temperature. Then, suspensions were evaluated with respect to their rheology by means of viscosity measurements (Brookfield, Model LVDV-Ⅱ+P, Shanghai, China).

### 2.3. Rheological Measurement

A concentric cylinder viscometer was applied to measure the rheological properties of the SiC dispersion (Brookfield, Model LVDV-Ⅱ+P). A thermostatic system was used along with the experiment, and the temperature was kept at 21 °C. In the flow test, the shear rates ranged from 0 to 200 s^−1^ with 50 step intervals and a wait of 2 s before each shear rate change. All rheological measurements were pre-sheared for 1 min at 100 s^−1^ followed by 30 s at a 0 s^−1^ shear rate to eliminate any effects owing to the loading of samples.

### 2.4. Adsorption Equilibrium of Dispersants on SiC Powder

The adsorption performance of PVP was investigated by the following method: a suspension having a solids content of 20 vol.% with a dispersant of different concentrations (according to the weight of the powder) was centrifuged at 9000 rpm to obtain SiC particles from the suspension. The obtained powder was dried in a thermostatic blast oven at 100 °C, and the weight of the adsorbed PVP on the particles was measured via a thermogravimetric technique (Q50, TA Instruments Ltd., Crawley, UK).

### 2.5. FTIR and XPS Measurements

The interaction of the dispersant on the surface of SiC particles was studied in a 400–4000 cm^−1^ region by Fourier transform infrared spectroscopy (Nicolet 6700, Thermo Electron Scientific Instruments, Shanghai, China). The powder was placed in a sample box, which was then placed in a diffuse reflection accessory. To improve the signal-to-noise ratio, the angle of the mirror was adjusted. The infrared spectroscopy of the silicon carbide powder that adsorbed PVP was collected using 128 scans with a background of pure silicon carbide powder at a resolution of 2 cm^−1^. In addition, the chemical properties of the silicon carbide particle surface were measured by XPS (ESCALAB 250Xi, Thermo Fisher Scientific, Shanghai, China).

## 3. Results and Discussion

### 3.1. Characterization of the Silicon Carbide Powder

**Figure 2:**
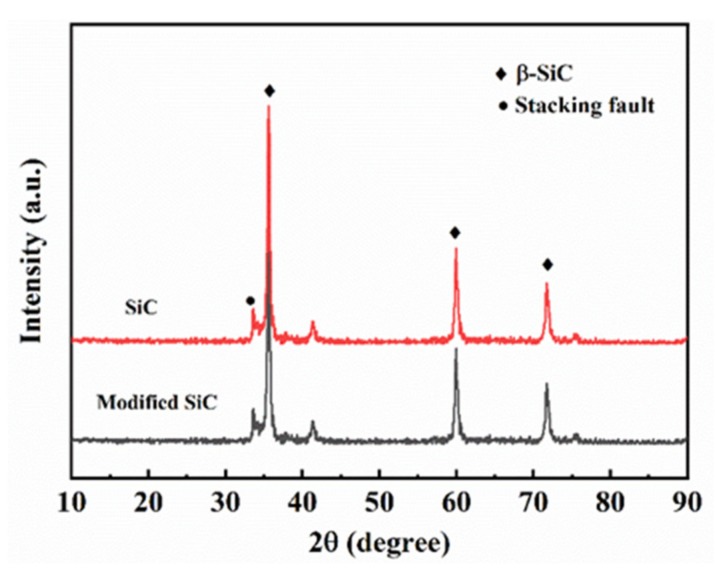
X-ray diffraction (XRD) patterns of the silicon carbide (SiC) powder and PVP-modified SiC powder.

**Figure 3:**
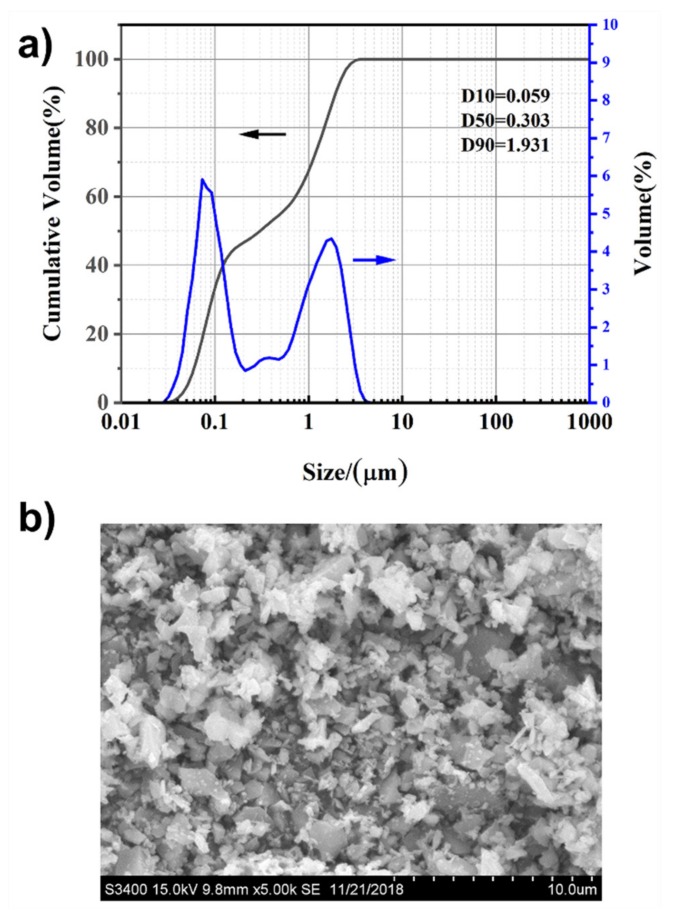
Particle size distribution of the as-received SiC powder (**a**) and scanning electron microscopy (SEM) image of the as-received SiC powder (**b**).

The XRD peak (Figure 2) of the silicon carbide powder confirmed the existence of only the β–SiC cubic sphalerite crystal structure, and PVP modified method does not alter the crystal structure of SiC. The particle size measurement and SEM results of the initial SiC particles are shown in Figure 3. The size distribution of SiC powder was distinctly bimodal, with a slender peak at 0.1 µm and a broad peak at 1 µm. The particle size was 0.303 µm at D50, and the particle size value provided by the manufacturer was 0.5 µm; multiple size analyses were conducted to confirm data accuracy and repeatability. From the graph above, we can substantially see that no particles having a size beyond these two ranges were detected. The SEM results were basically consistent with the particle size data; most of the particles were ~1 µm, and the fines were easily observed. Scanning electron microscopy (SEM) images also showed that the morphology of these particles was irregular and "plate-like" in nature.

### 3.2. Rheological Measurement

**Figure 4:**
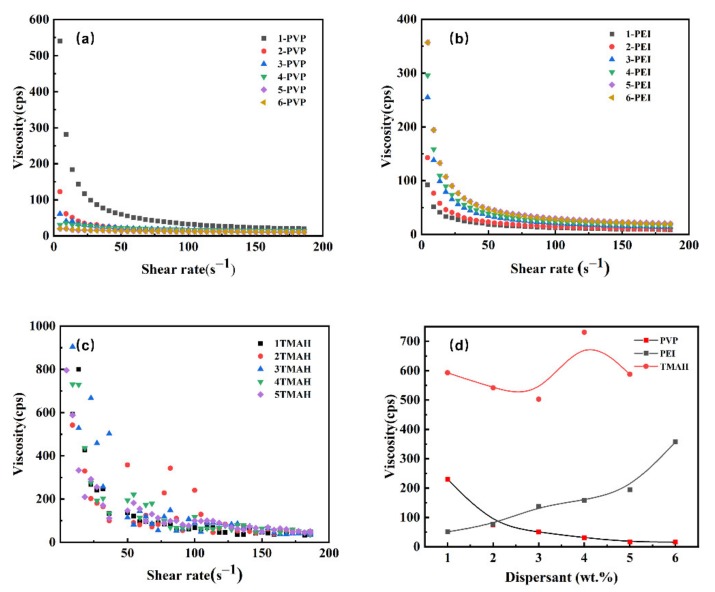
Viscosity as a function of the shear rate of SiC slurries with 20 vol.% solids loading under different concentrations of dispersants (**a**–**c**), and the viscosity of different dispersants at the determined shear rate with 10 s^−1^ (**d**).

The rheological behaviors of the suspensions by adding different concentrations of dispersant were studied at a constant solids content of 20 vol.%. The main focus was two properties: dynamic viscosity and fluid form. In Figure 4a–c, under different dispersants, the shear viscosity was plotted as a function of shear rate. As expected, the suspension at 20 vol.% had a very high shear viscosity. This was obviously the result of its high solids content, leading to a large increase in particle-particle interaction that gave the slurry a higher yield stress value. Figure 4a shows that, after the addition of PVP, the viscosity of the suspension decreased with an increasing concentration of added PVP, because the saturated amount of PVP was high. Thus, until the addition of PVP reached 5 wt.%, saturated adsorption was achieved. A dispersant exhibits good dispersion when the dispersion has quite low viscosity at the saturated adsorption content. Figure 4a,b show that the viscosities of the dispersions vary, and the dispersion with an addition of 5 wt.% PVP can obtain the lowest viscosity (17 mPa⋅s at 25 s^−1^), while the addition of 1 wt.% PEI can obtain a low viscosity (27 mPa⋅s at 25 s^−1^). However, it is worth noting that a suspension with added PEI decreases the viscosity of the suspension as the amount of PEI increases. This was probably because the saturated adsorption amount of the PEI molecules was low; therefore, adding too much PEI increased the viscosity owing to polymer chain bridging. Figure 4c confirms that TMAH was not an appropriate additive for dispersing SiC powder in ethanol. This was because of the difficulty in ionizing in ethanol, and it was not effective to achieve electrostatic repulsion stability [25]. The data in Figure 4d show the relative efficiencies of these dispersants at the determined shear rate (10 s^−1^); PVP and PEI can be identified as the efficient dispersant dispersing nano-SiC in ethanol solvent.

**Figure 5:**
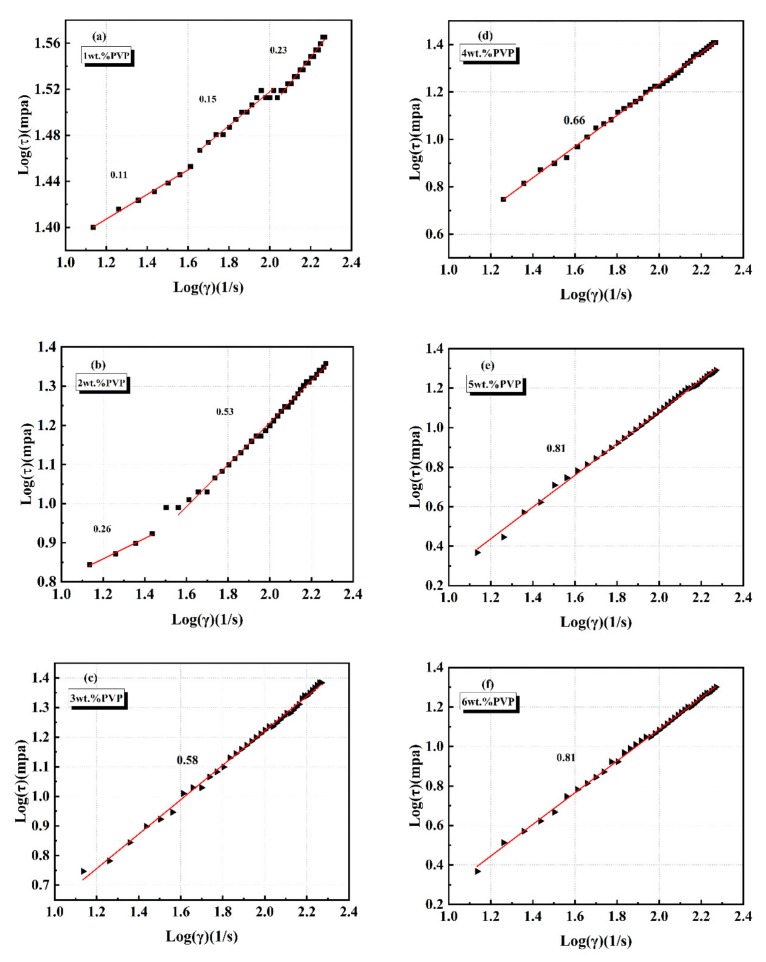
Double logarithmic power law plot at (**a**)1wt.%, (**b**) 2wt.%, (**c**) 3wt.%, (**d**) 4wt.%, (**e**) 5wt.%, (**f**) 6wt.% of dispersant for PVP-stabilized suspensions (at 20 vol.%). The viscosity data are presented in the inset of main figure.

As slurry with the lowest viscosity was obtained by using PVP in the experiments, in the next step, we mainly chose PVP as a dispersant to study its effect on the flow behavior of the SiC suspensions. The rheological behavior of the dispersion can be derived from the relationship between applied shear stress and shear rate [38,39,40,41]. The relation between shear stress and shear rate can be described by a power–law relation (Equation (2)), while Equation (3) was obtained by taking the logarithm of both sides of Equation (2) [42,43].

(2) $$\tau =b{\dot{\gamma }}^{n}$$

(3) $$\log\tau =\log b+n\log\dot{\gamma }a=1,$$

where *τ* is the shear stress; $\dot{\gamma }$ is the applied shear rate; and b and n are the parameter and the power-law index constants, respectively. With *n* < 1, the suspension was described as a shear-thinning fluid; when *n* = 1, a Newtonian-like fluid was described; and when *n* > 1, shear-thickening was described. We intensively studied the effect of PVP as a dispersant on the flow index of the suspension. The flow index was the slope of the above relation (Equation (2)) in a logarithmic plot. Figure 5 shows the variation of the flow index of the 20 vol.% silicon carbide suspension with the increase of additional PVP (see Figure 5a–f). The suspension had multi-flow index at low amounts of dispersant (see Figure 5 a,b), indicating that the shear thinning effect of the suspension under external force was obvious. In addition, as the applied shear rate increased, the flow index gradually increased. However, when the dispersant content was greater than 3wt.%, the system exhibited a single flow index over the entire shear rate range of the experiment (see Figure 5c–f), indicating that the system exhibited stable rheological behavior. In particular, when the value of the flow index was close to 1, it reflected quasi-Newtonian flow behavior [44]. When the appropriate amount of PVP was added, the suspension had a single flow factor with the changed shear rate, and the value approached 1. This indicated that the suspension was in the form of a stable quasi-Newtonian fluid, compared with a suspension with a multi-flow index. Thus, PVP with an optimum dose could produce a stable colloidal dispersion, which could be reflected by a unique slope profile and quasi-Newtonian properties, as shown in Figure 5e,f, and was determined by a flow factor value approaching 1. Regarding PVP, the literature reports that the carbonyl of PVP was beneficial for adsorbing on alkaline surfaces. Therefore, PVP was probably adsorbed via a perpendicular conformation with pyrrolidone as the adsorbing head, and the hydrogen atoms in the chain promoted absorption on the acidic ceramic surface [45,46,47].

### 3.3. Adsorption Mechanism of Dispersants on SiC Powder

Different solid-adsorbate systems gave different adsorption models. Adsorption models have monolayer and multilayer adsorption. The Langmuir isotherm model is the most common single-layer adsorption equation. The single-layer isotherm curve can be expressed as follows [48]:

(4) $$q_{e}=\frac{q_{m}bC_{e}}{\left(1+bC_{e}\right)}$$

A plot of *C*_e_/*q*_e_ versus *C*_e_ gives a straight line written as follows:

(5) $$\frac{C_{e}}{q_{e}}=\frac{1}{bq_{m}}+\frac{C_{e}}{q_{m}}$$

where *q*_e_ (mg/g) is the balanced adsorption content, b is a constant, *q*_m_ is the adsorption limit, and *C*_e_ (g/L) is the amount of PVP at saturation. The Freundlich isotherm curve is a classical multilayer adsorption equation that can be expressed as follows [48]:

(6) $$q_{e}=bC_{e}^{k}$$

**Figure 6:**
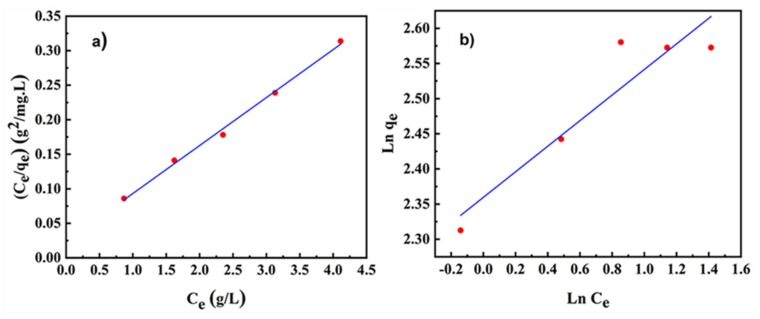
Langmuir model fitting (**a**) and Freundlich model fitting (**b**) of the adsorption of PVP on SiC.

Equation (7) can be obtained by taking the logarithm on both sides of Equation (6):

(7) $$\ln q_{e}=k\ln C_{e}+\ln b$$

where *q*_e_ (mg/g) is the equilibrium adsorption capacity, *C*_e_ (g/L) is the concentration of the solute at equilibrium, b is the Freundlich parameter, and k is the adsorption constant. Figure 6 shows the fitted curves of the Langmuir and Freundlich models. As shown in Figure 6b, the curve obtained from the experimental data deviated from the curve fitted by the Freundlich adsorption model. Figure 6a shows that the curve obtained from the experimental data conformed to the curve plotted by the Langmuir model, which confirms that the adsorbed PVP molecule interacted with only one site and did not interact with other adsorbed molecules and formed a monolayer to some extent. The above results were also consistent with the viscosity results in Figure 4a.

### 3.4. FTIR and XPS Characterization

**Figure 7:**
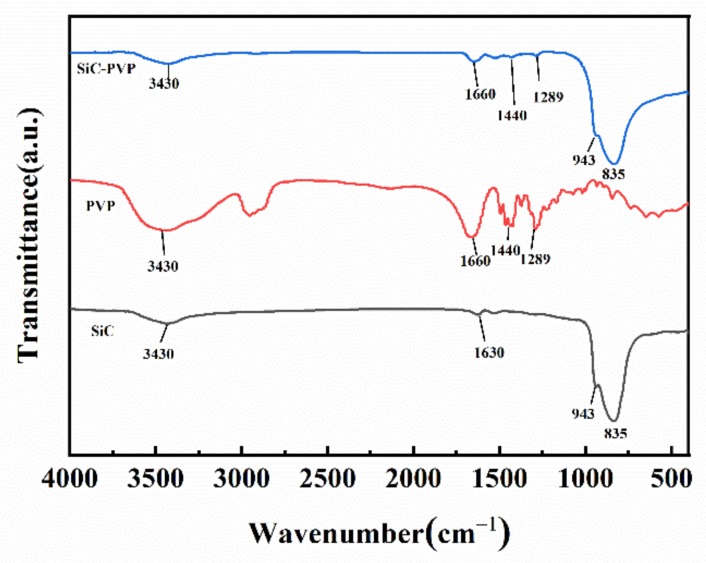
Fourier transform infrared spectroscopy (FTIR) spectra of SiC, PVP, and PVP-adsorbed SiC.

We further studied the chemical information of PVP molecules on the SiC surfaces. Figure 7 shows the interaction between SiC and PVP by FTIR spectroscopy. The infrared absorption band of SiC in Figure 7 had peak values of 3430, 1630, 943, and 835 cm^−1^, which were caused by OH stretching, OH stretching from water vapor by physical adsorption, Si–O–Si, and Si–C stretching vibration, respectively [45,46,49]. IR absorptions of SiC with adsorbed PVP primarily occurred at 3430, 1660–1289, and 835 cm^−1^. The peaks at 1630–1289 cm^−1^ originated from the adsorbed PVP and the 1660–1289 cm^−1^ peaks that emerged in the spectrum of PVP-modified SiC were attributed to the stretching of C–N and C=O. These peaks confirmed that PVP was successfully adsorbed upon the SiC surface [45,46].

**Figure 8:**
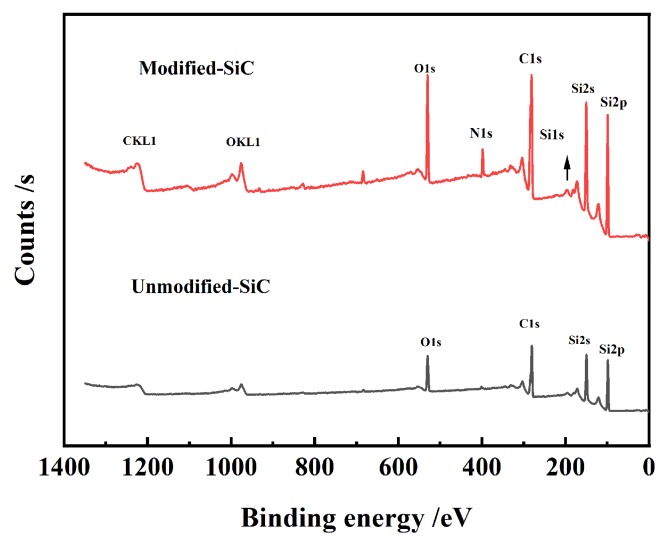
X-ray photoelectron spectroscopy (XPS) spectra of as-received SiC and PVP-adsorbed SiC particles.

**Figure 9:**
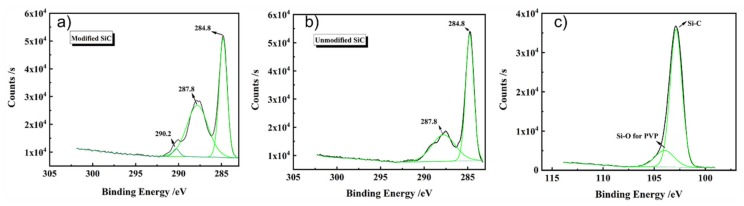
High-resolution XPS spectra: (**a**) C 1s of PVP-adsorbed SiC powder, (**b**) C 1s of unmodified SiC powder, and (**c**) Si 2p of PVP-adsorbed SiC powder.

The chemical properties of the PVP-modified SiC were further investigated by XPS characterization. It can be seen from the data in Figure 8 that the XPS spectra of the SiC powder and modified SiC powder that the XPS spectra exhibited the peaks of C KLL, O KLL, C 1s, O 1s, O 2s, N 1s, Si 2s, and Si 2p. Nitrogen, as the unique element of PVP, existed on the SiC powder surface, demonstrating that PVP had been adsorbed on the SiC surface. Figure 9a,b show the C 1s spectra of PVP-modified SiC powder and unmodified SiC powder. The peak appearing at 290.2 eV corresponded to C=O, which further indicated that PVP had been grafted onto the SiC surface compared with that of the pure silicon carbide powder. Figure 9c shows the Si 2p spectra of PVP-modified SiC particles. From Figure 9c above, we can see that the two peaks at 103.18 eV and 104.38 eV were related to Si–C and Si–O, respectively [49]. The existence of Si–OH confirmed the presence of an active site for the adsorption of PVP on the surface of silicon carbide, and hydrogen bonds could be formed between the surface of the SiC and PVP molecules.

### 3.5. Maximum Ceramic Loading of the PVP Stabilized Systems

**Figure 10:**
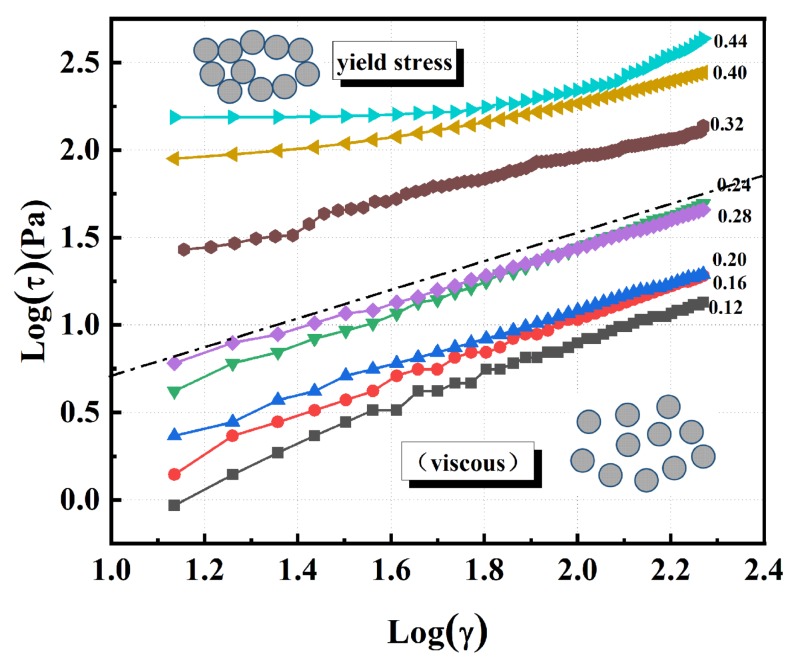
The logarithmic plot of the power law for 5 wt.% PVP stabilized suspensions. The inset presents an illustration of the particle distribution model.

**Figure 11:**
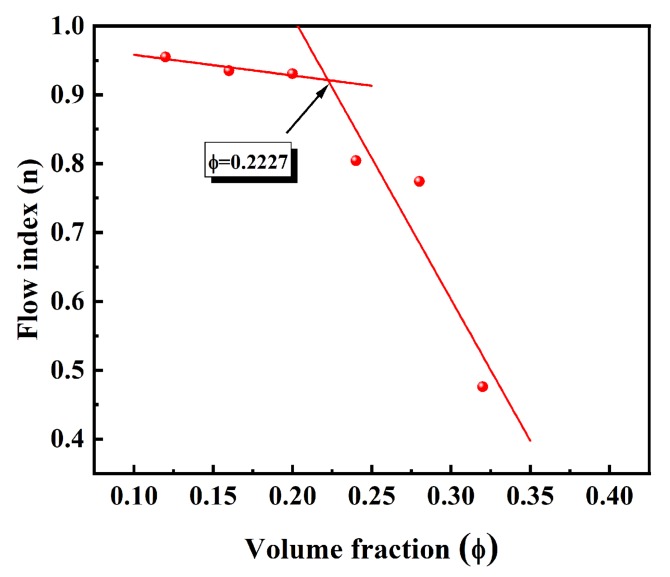
The plot of the flow index factor as a function of ceramic content for PVP-stabilized suspensions at an optimum PVP amount.

The rheological behavior of the ceramic suspension is an important aspect of colloidal ceramic formation. Figure 10 shows suspensions with an optimal amount (5 wt.%) of dispersant in suspension produced increased ceramic content, and its flow behaviors were studied. After the volume content of the suspensions exceeded 32 vol.%, the single flow index factor of the suspensions had a multi flow index, indicating that a soft agglomeration structure began to form inside the suspension, which was easily affected by the leading role of the applied shear force. Furthermore, we can see from Figure 11 that, when the volume loading was greater than 22.27 vol.%, the flow index of the suspension began to mutate and the suspension began to change from a Newtonian-like fluid to a shear-thinning fluid. The flow factor method showed that PVP was a dispersant that can simultaneously produce a wide single flow index and a quasi-Newtonian region, confirming that a PVP-adsorbed SiC suspension has excellent rheological properties. Finally, we explored the issue of the maximum solids loading of the suspensions with PVP as the dispersant. Two models were used for the estimation [50]:

(8) $$\frac{\eta_{nf}}{\eta_{bf}}=1+2.5\phi$$

(9) $$\frac{\eta_{nf}}{\eta_{bf}}={\left(1-\frac{\phi_{}}{\phi_{c}}\right)}^{-[\eta ]\phi_{c}}$$

**Figure 12:**
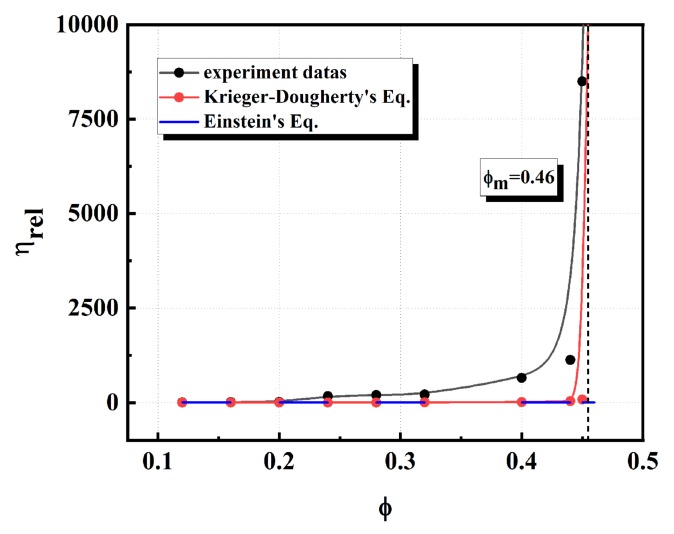
The relative viscosity of the SiC slurry as a function of solids loading determined by different models.

In the Einstein relation (Equation (8)), *η_nf_* and *η_bf_* measure the viscosity of the suspension and suspending medium, respectively, and *ϕ* is the solids loading of the suspension. The equation for the Krieger-Dougherty (K-D) model (Equation (9)) describes the loading dependence of the viscosity of suspensions, where *ϕ_c_* is the maximum volume loading of the suspension (the viscosity of the suspension raised to infinity) and *η* is the intrinsic viscosity, which could reflect the characteristics of particle agglomeration, being 2.5 for rigid particles [51]. As shown in Figure 12, we recorded the viscosity values of the suspension under different volume loadings. We also plotted the K-D model curve and the Einstein viscosity curve. The results of Figure 12 illustrate that the K-D model fitted well with the experimental data at a higher volume fraction and can be used to predict the maximum solid phase content. In contrast, the Einstein model fits well only at low concentrations. From the K-D model, we can obtain a maximum volume content of 46 vol.% SiC slurry.

## 4. Conclusions

The main goal of the current study was to determine the optimal dispersing agents with different chemical properties to disperse silicon carbide powder in an organic solvent. The three dispersants were amphiphilic, acidophilic, and alkaliphilic. As a result, PVP and PEI were found to be suitable for achieving good dispersion of nano-SiC in ethanol. In addition, the rheological index factor was put forward to quantitatively characterize the rheological properties of the slurry. The single index factor reflected the good stability of the suspension. Further investigation of the adsorption mechanism was discussed.

The adsorption mechanism of PVP-modified silicon carbide powder was first investigated. The results obtained revealed that the adsorption isotherm equation was basically consistent with the Langmuir isotherm model. FTIR and XPS data indicated that PVP successfully modified the surface of the silicon carbide particles.

In the final part of the experiments, the maximum ceramic loading of PVP-stabilized systems was investigated. We found that using the K-D model to predict the maximum solids content of the slurry at high solids levels was more reliable compared with the other models. The theoretical maximum solids content of the silicon carbide suspension was 46 vol% with micro- and nanoparticles stabilized by PVP.
